# Detection and Quantification of ADP-Ribosylated RhoA/B by Monoclonal Antibody

**DOI:** 10.3390/toxins8040100

**Published:** 2016-04-01

**Authors:** Astrid Rohrbeck, Viola Fühner, Anke Schröder, Sandra Hagemann, Xuan-Khang Vu, Sarah Berndt, Michael Hust, Andreas Pich, Ingo Just

**Affiliations:** 1 Institute of Toxicology, Hannover Medical School, Carl-Neuberg-Str. 1, 30625 Hannover, Germany; schroeder.anke@mh-hannover.de (A.S.); hagemann.sandra@mh-hannover.de (S.H.); xuan-khang.vu@stud.mh-hannover.de (X.-K.V.); berndtsarah@web.de (S.B.); pich.andreas@mh-hannover.de (A.P.); just.ingo@mh-hannover.de (I.J.); 2 Biotechnology and Bioinformatics, Department of Biotechnology, Institute for Biochemistry, Technische Universität Braunschweig, Spielmannstr. 7, 38106 Braunschweig, Germany; v.fuehner@tu-braunschweig.de (V.F.); m.hust@tu-bs.de (M.H.)

**Keywords:** C3-transferase, ADP-Ribosyltransferase, ADP-ribosylated RhoA

## Abstract

*Clostridium botulinum* exoenzyme C3 is the prototype of C3-like ADP-ribosyltransferases that modify the GTPases RhoA, B, and C. C3 catalyzes the transfer of an ADP-ribose moiety from the co-substrate nicotinamide adenine dinucleotide (NAD) to asparagine-41 of Rho-GTPases. Although C3 does not possess cell-binding/-translocation domains, C3 is able to efficiently enter intact cells, including neuronal and macrophage-like cells. Conventionally, the detection of C3 uptake into cells is carried out via the gel-shift assay of modified RhoA. Since this gel-shift assay does not always provide clear, evaluable results an additional method to confirm the ADP-ribosylation of RhoA is necessary. Therefore, a new monoclonal antibody has been generated that specifically detects ADP-ribosylated RhoA/B, but not RhoC, in Western blot and immunohistochemical assay. The scFv antibody fragment was selected by phage display using the human naive antibody gene libraries HAL9/10. Subsequently, the antibody was produced as scFv-Fc and was found to be as sensitive as a commercially available RhoA antibody providing reproducible and specific results. We demonstrate that this specific antibody can be successfully applied for the analysis of ADP-ribosylated RhoA/B in C3-treated Chinese hamster ovary (CHO) and HT22 cells. Moreover, ADP-ribosylation of RhoA was detected within 10 min in C3-treated CHO wild-type cells, indicative of C3 cell entry.

## 1. Introduction

The C3 exoenzyme from *Clostridium botulinum* ADP-ribosylates specifically RhoA, B, and C at amino acid 41 (asparagine) thereby inactivating it [1]. RhoA belongs to a family of small GTPases that are active in GTP-bound and inactive in GDP-bound form and are involved in regulation of cell division, growth, migration, and neural development. RhoA also serves as a key mediator between extracellular signals and cytoskeleton rearrangement [2]. Moreover, incubation of neurons with C3 prevents growth cone collapse and induces axonal growth [3]. C3 is a simple exoenzyme that lacks specific binding and translocation domain of intracellular acting bacterial protein toxins. Nevertheless, C3 is efficiently internalized into different cell types [4] as indicated by ADP-ribosylation of intracellular RhoA. The widely used methods for detection of ADP-ribosylated RhoA are (i) the analysis of the migration behavior of modified RhoA in sodium dodecyl sulfate-polyacrylamide gel electrophoresis (SDS-PAGE) and subsequent Western blot for RhoA detection, or (ii) the conventional radioactive sequential-[^32^P]-ADP-ribosylation assay. The SDS-PAGE/Western blot method is a direct way of detection but analysis of ADP-ribosylated RhoA is difficult due to the slight difference in molecular weight of 541 Da compared to the unmodified RhoA. ADP-ribosylated RhoA migrates to a higher apparent molecular weight in SDS-PAGE [5] and this gel-shift assay is difficult to interpret and may lead to inconsistent results. Inadequate separation of proteins in SDS-PAGE prevents the migration of ADP-ribosylated RhoA to the slight higher molecular weight, so that the ADP-ribosylated RhoA cannot be distinguished from the unmodified RhoA. Considering these limitations, it is recommended to perform a second method to confirm the results of the gel-shift assay. The radioactive sequential-[^32^P]-ADP-ribosylation assay is often used as an additional method. Since [^32^P]-NAD is not cell permeable [6] this assay can only be used to label Rho proteins in cell lysates. Therefore, this method only proves how much unlabeled Rho is still present in the C3-treated cells and, thus, provides only an indirect evidence of the cellular uptake of C3. A specific antibody against ADP-ribosylated RhoA would be a great advantage for better monitoring of C3-mediated modification of RhoA. Therefore, we generated an antibody that is specific for ADP-ribosylated RhoA/B and we analyzed this antibody both in cell-free and cellular system. This ViF140_A1-hFc antibody recognizes only ADP-ribosylated RhoA/B and allows quantitative monitoring of C3-mediated ADP-ribosylation of Rho. Additionally, this new antibody allows an immunocytochemical application in cell biology.

## 2. Results

### 2.1. Detection of C3-Catalyzed ADP-Ribosylation of RhoA in CHO Cells within 10 min

**Figure 1:**
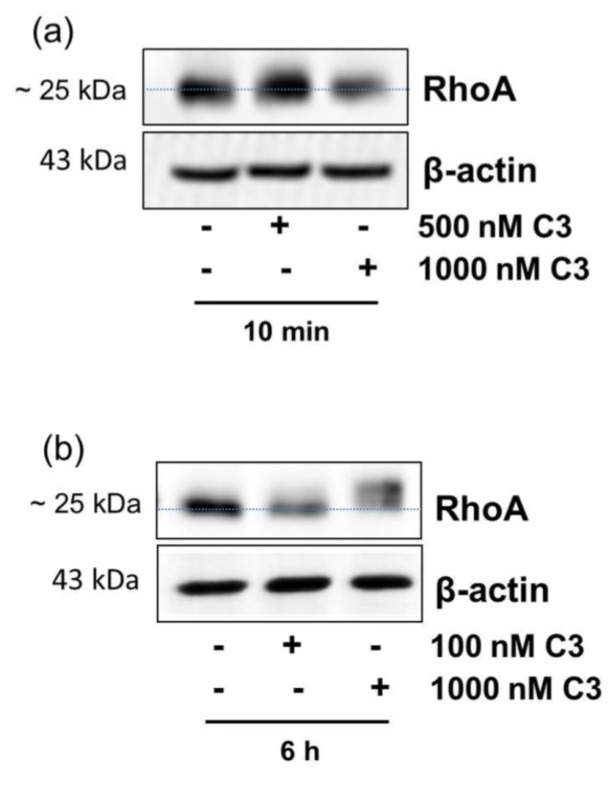
Detection of C3-catalyzed ADP-ribosylation of RhoA in Chinese hamster ovary (CHO) cells within 10 min. CHO cells (**a**) and hippocampal HT22 cells (**b**) were treated with increasing concentrations of C3 for indicated time points at 37 °C. Cells were lysed and submitted to Western blot analysis against RhoA and β-actin.

C3-catalyzed ADP-ribosylation of RhoA in cell lysates was detected by altered migration behavior of RhoA in SDS-PAGE/Western blotting. In CHO cells C3 caused ADP-ribosylation of RhoA within 10 min (Figure 1a). Unfortunately, the altered migration behavior of RhoA in gel-shift assay is often ambiguous in CHO cells, so that a different approach is necessary for a clear interpretation. The hippocampal HT22 cell line was used as control because it is susceptible to C3 and the RhoA shift is more precise [7,8]. C3-catalyzed ADP-ribosylation of RhoA in HT22 cells is delayed and was analyzed after 6 h (Figure 1b).

### 2.2. Sequential [^32^P]-ADP-Ribosylation Confirmed Uptake of C3 within Minutes

**Figure 2:**
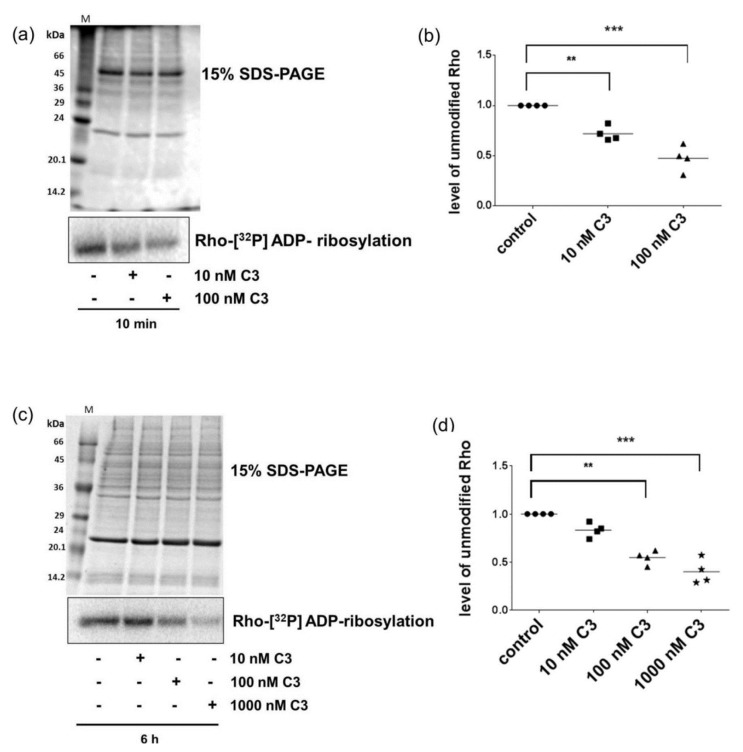
Sequential [^32^P]-ADP-ribosylation of Rho. CHO cells (**a**) and HT22 cells (**c**) were treated with different C3 concentrations for indicated time points. Subsequently, cell lysates were incubated with 1 µM C3 and 1 µCi [^32^P]-NAD in 20 µL of buffer containing 50 mM 4-(2-hydroxyethyl)-1-piperazineethanesulfonic acid (HEPES) (pH 7.3), 10 mM MgCl_2_, 10 mM dithiothreitol, 10 mM thymidine, and 10 µM nicotinamide adenine dinucleotide (NAD) at 37 °C for 30 min. The reaction was terminated by addition of Laemmli sample buffer and then incubated at 95 °C for 10 min. Samples were resolved by 15% sodium dodecyl sulfate-polyacrylamide gel electrophoresis (SDS-PAGE), and the [^32^P]-ADP-ribosylated Rho was analyzed by phosphorimaging. Representative phosphorimaging of [^32^P]-ADP-ribosylated Rho and the Coomassie brilliant blue stained SDS-PAGE gels as loading control are presented (*n* = 4). Molecular masses are indicated in kDa. M = Marker SDS7 (Sigma-Aldrich Chemie GmbH, Taufkirchen, Germany); and (**b**,**d**) diagrams depict cellular level of unmodified Rho (● control, ■ 10 nM C3, ▲ 100 nM C3, ★ 1000 nM C3). Cellular level of unmodified Rho was determined by densitometrically quantification of intensity of sequential [^32^P]-ADP-ribosylated Rho using Kodak software 1D, Version 3.5. The signal intensity of [^32^P]-ADP-ribosylated Rho from untreated control cells were set as 1. Statistical differences were determined by two-sided Student’s *t* test (* *p* ≤ 0.05; ** *p* ≤ 0.01; *** *p* ≤ 0.001).

To validate that C3 was in fact internalized into the CHO cells within this short time period, a sequential radioactive ADP-ribosylation assay was performed based on the notion that ADP-ribosylation of Rho in intact cells prevents the second radioactive ADP-ribosylation of Rho in the cell lysates. To this end, cell lysates from C3-treated cells (40 µg) were subjected to *in vitro* [^32^P]-ADP-ribosylation with C3. Subsequently, the samples were separated by SDS-PAGE and the extent of [^32^P]-ADP-ribosylation of Rho was determined. As shown in Figure 2a, C3 uptake into CHO cells within 10 min was confirmed by reduced [^32^P]-ADP-ribosylation of Rho (Figure 2a). ADP-ribosylation of Rho was concentration dependent, with the strongest effect at a C3 concentration of 100 nM (Figure 2b). Thus, such low C3 concentration was sufficient to ADP-ribosylate Rho in intact CHO cells within minutes. This finding is in strong contrast to previous reports on other cell lines. In HT22 cells Rho ADP-ribosylation was observed only partially after 6 h (Figure 2c), but also in a concentration-dependent manner (Figure 2d).

### 2.3. Mass Spectrometric Detection of ADP-Ribosylated RhoA

**Figure 3:**
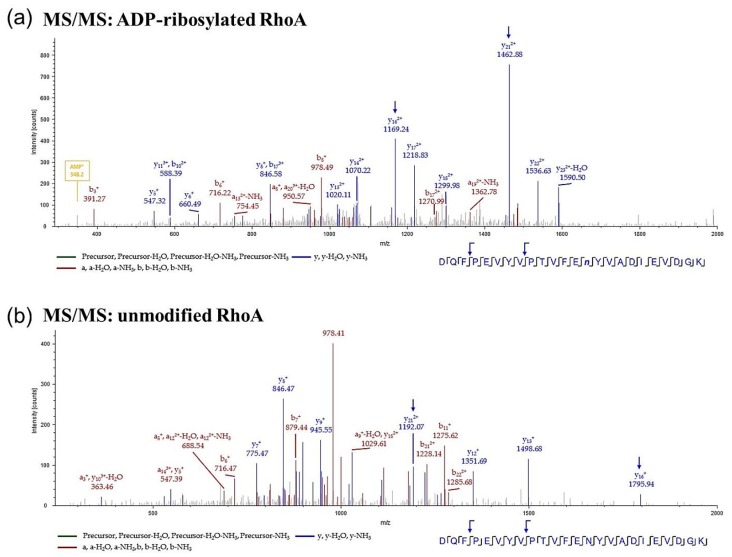
Mass spectrometric detection of ADP-ribosylated RhoA in CHO cells. CHO cells were treated with 500 nM C3 or C3-E174Q for 10 min. Untreated cells served as control. Subsequently, cell lysates were separated by 15% SDS-PAGE and stained with Coomassie brilliant blue. The samples of 20–30 kDa bands were digested with trypsin and peptides were subjected to mass spectrometry analysis. A MS/MS spectrum of the analyzed RhoA peptide is shown. (**a**) Identification of ADP-ribosylated RhoA; and (**b**) unmodified RhoA, blue arrows show specific *y*-ions/fragments of the modified or unmodified peptide.

Specific ADP-ribosylated asparagine and arginine residues can be identified by mass spectrometry (MS) [9,10,11] and C3-dependent Rho-ADP-ribosylation in HT22 cells has already been reported by MS [12]. Therefore, cell lysates from C3-treated (500 nM C3 or C3-E174Q, 10 min) CHO cells were separated by SDS-PAGE and the proteins within 20–30 kDa range were tryptically digested. The resulting peptides were analyzed by liquid chromatography-mass spectrometry (LC-MS). The ADP-ribosylated RhoA peptide showed a precursor mass shift of 541 Da and was identified by its *y*- and *b*-ion series. The ADP ribosylation was directly detected at asparagine-41 (Figure 3a). Untreated CHO cells and cells treated with the enzyme-deficient C3-E174Q mutant served as control. The LC-MS approach confirmed the very fast ADP-ribosylation of RhoA in CHO cells. RhoA ADP-ribosylation was not detected in C3-E174Q-treated or in untreated CHO cells (Figure 3b, Table 1).

### 2.4. Detection and Quantification of Adp-Ribosylated Rhoa/B by Monoclonal Antibody

**Figure 4:**
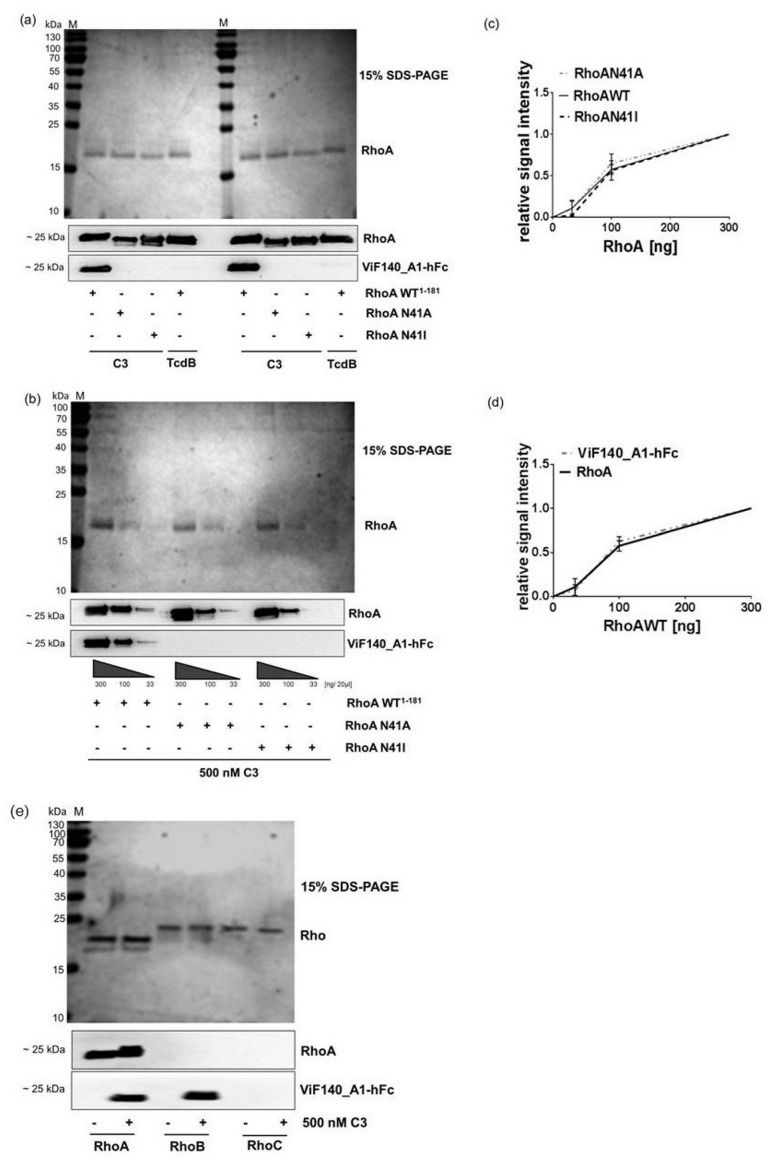
Detection and quantification of ADP-ribosylated recombinant RhoA by monoclonal antibody. (**a**) Wild-type (RhoA WT) and non-ADP-ribosylatable RhoA (RhoA N41A and RhoA N41I) were ADP-ribosylated with 1000 nM C3 or glycosylated with TcdB. Samples were probed with antibodies recognizing Rho (RhoA) or ADP-ribosylated Rho (ViF140_A1-hFc). The displayed Western blots (in duplicate) are representative from three independent experiments; (**b**) decreasing amounts of C3-treated samples of experiment (**a**) were analyzed with RhoA and ADP-ribosylated RhoA/B recognizing antibodies; (**c**) densitometric analysis of (**b**) RhoA antibody is shown; (**d**) densitometric analysis of (**b**) ADP-ribosylated Rho antibody is shown; and (**e**) recombinant wild-type RhoA, RhoB, and RhoC were ADP-ribosylated with 500 nM C3. Samples were probed with antibodies recognizing Rho (RhoA) or ADP-ribosylated Rho (ViF140_A1-hFc). Exemplary, Coomassie brilliant blue stained SDS-PAGE gel shows the same amounts of Rho WT, RhoA N41A and RhoA N41I. Molecular masses are indicated in kDa. M = PageRuler prestained protein ladder (Thermo Fisher Scientific Inc., Waltham, MA, USA).

The SDS-PAGE/Western blot and sequential radioactive ADP-ribosylation assay described above did not result in clear-cut data. Western blot assays generally lack the sensitivity to measure low levels of ADP-ribosylated Rho with accuracy and reliability and quantification of ADP-ribosylated Rho varies and is not reproducible. Quantification of ADP-ribosylated Rho using sequential radioactive ADP-ribosylation assay is difficult because of large variability between the assays. Furthermore, a non-radioactive detection method would be helpful and cost-effective. Therefore, a monoclonal antibody recognizing ADP-ribosylated Rho has been developed as described previously [13,14]. This new antibody specifically recognized exclusively ADP-ribosylated recombinant wild type RhoA but not unmodified RhoA or glycosylated RhoA formed by *Clostridium difficile* Toxin B (TcdB) (Figure 4a). The antibody was tested for quantification of ADP-ribosylated RhoA. To this end, decreasing amounts of either ADP-ribosylated wild-type RhoA (RhoAWT) or non-ADP-ribosylatable mutated RhoA (RhoA N41A, RhoA N41I) plus C3 were analyzed with the new antibody in Western blotting (Figure 4b). Signal intensities in Western blot clearly correlated with decreasing amounts of wild type ADP-ribosylated RhoA (Figure 4c,d) whereas non-ADP-ribosylated RhoA was not recognized. To check whether the new antibody against ADP-ribosylated Rho only recognized RhoA but also RhoB or RhoC, we investigated ADP-ribosylated recombinant RhoB and RhoC in Western blot analysis. As shown in Figure 4e, the antibody detects ADP-ribosylated RhoA and RhoB but not RhoC. This is not surprising due to the high sequence identity of Rho proteins. Especially, the amino acids adjacent to asparagine 41 are identical in these Rho-GTPases with the exception of position 43. Amino acid residue 43 in RhoA/B is valine, whereas it is isoleucine in RhoC. The findings suggest that a divergence between RhoA/B and RhoC at residue 43 is important for recognizing of ADP-ribosylated antibody. These data clearly suggest that the generated antibody is specific to ADP-ribosylated RhoA/B.

**Figure 5:**
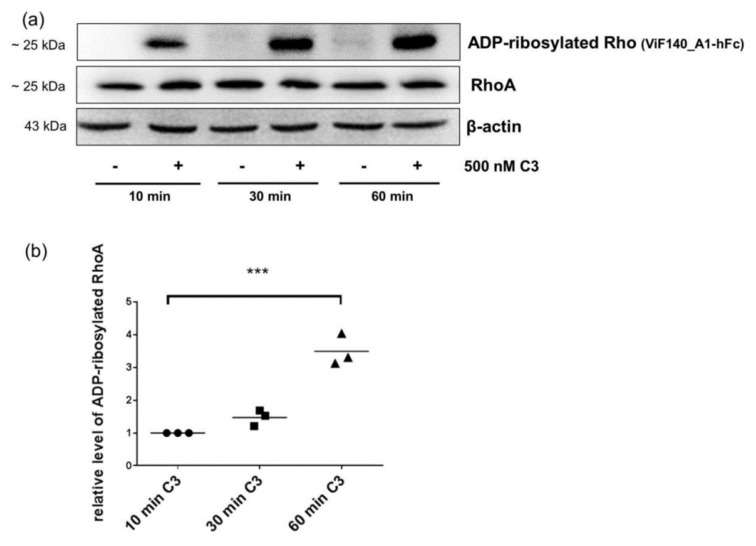
Detection and quantification of ADP-ribosylated RhoA/B by monoclonal antibody in CHO cells. CHO cells were treated with 500 nM C3 for indicated time points at 37 °C. (**a**) Cells were lysed and submitted to Western blot analysis against ADP-ribosylated RhoA/B or total RhoA and β-actin; and (**b**) the diagram depicts the cellular level of ADP-ribosylated RhoA/B (● 10 min C3, ■ 30 min C3, ▲ 60 min C3). The cellular level of ADP-ribosylated RhoA/B was determined by densitometric quantification of intensity of detected modified RhoA/B using Kodak software and adjusted to the corresponding β-actin signal. The signal intensity of ADP-ribosylated RhoA/B from 10 min C3-treated cells was set as 1. Statistical differences were determined by two-sided Student’s *t* test (*** *p* ≤ 0.001).

As shown in Figure 5a, ADP-ribosylated RhoA/B was in fact detected in CHO cells after 10 min of C3 treatment and the amount of ADP-ribosylated RhoA/B increased in a time-dependent manner (Figure 5b), whereas the level of total RhoA remains the same (Figure 5a).

**Figure 6:**
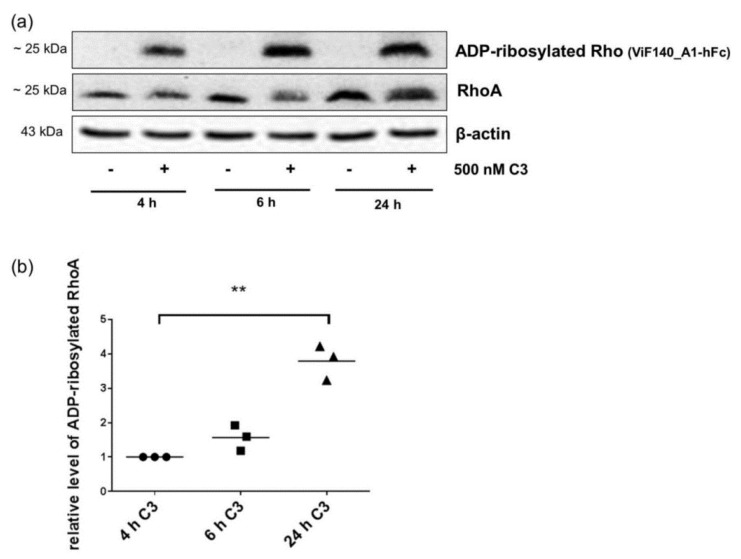
Detection and quantification of ADP-ribosylated RhoA/B by monoclonal antibody in hippocampal HT22 cells. (**a**) HT22 cells were treated with 500 nM of C3 for indicated time points at 37 °C. Cells were lysed and submitted to Western blot analysis against ADP-ribosylated RhoA/B or total RhoA and β-actin; and (**b**) the diagram depicts the cellular level of ADP-ribosylated RhoA/B (● 4 h C3, ■ 6 h C3, ▲ 24 h C3). The cellular level of ADP-ribosylated RhoA/B was determined by densitometric quantification of the intensity of detected ADP-ribosylated RhoA/B using Kodak software and adjusted to the corresponding β-actin signal. The signal intensity of ADP-ribosylated RhoA/B from 4 h C3-treated cells was set as 1. Statistical differences were determined by two-sided Student’s *t* test (** *p* ≤ 0.01).

In HT22 cells ADP-ribosylated RhoA/B was slightly detected after 4 h but clearly after 6 h (Figure 6a). The signal of ADP-ribosylated RhoA/B increased time dependently (Figure 6b). The intensity of ADP-ribosylated RhoA/B correlated well with the gel-shift assay (Figure 6a).

**Figure 7:**
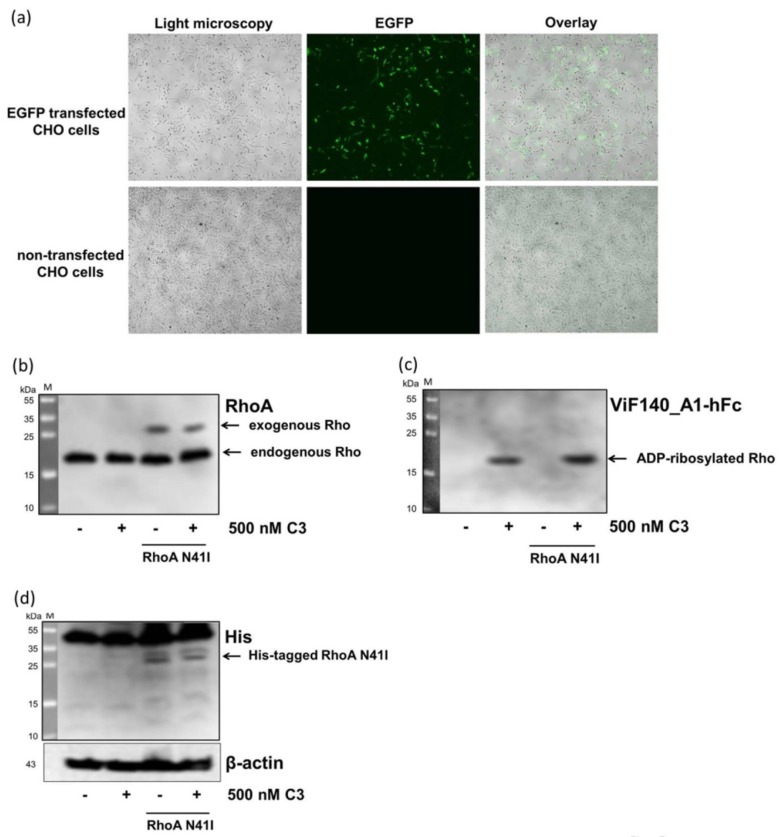
Detection of ADP-ribosylated RhoA/B by monoclonal antibody in RhoA N41I transfected CHO cells. (**a**) For determination of transfection effectiveness, cells were transfected with a pQE-TriSystem-EGFP vector encoding for enhanced green fluorescent protein (EGFP). Representative images of transfected CHO cells 48 h after transfection show cells in light microscopy, EGFP expression, and an overlay of both (4 × magnification). For Western blot analysis, CHO cells were transfected with RhoA N41I DNA plasmid or EGFP-coding plasmid with jetPrime for 4 h in the presence of serum, followed by treatment with 500 nM of C3 for 10 min. Cells were lysed and submitted to Western blot analysis against total RhoA (**b**) or ADP-ribosylated RhoA/B (**c**); His and β-actin (**d**). Molecular masses are indicated in kDa. M = PageRuler prestained protein ladder.

To confirm the specificity of the novel antibody against ADP-ribosylated RhoA/B, we transfected CHO cells with non-ADP-ribosylatable Rho N41I. The Rho N41I plasmid and the enhanced green fluorescent protein (EGFP) vector were transfected into CHO cells with relative low transfection efficiencies and low cytotoxic effects (Figure 7a), which was reflected by low protein expression (Figure 7b,d). However, as shown in Figure 7b, RhoA antibody discriminated between exogenous (30 kDa) and endogenous RhoA (25 kDa) in His-tagged–RhoA N41I-transfected CHO cells. Furthermore, the ADP-ribosylated RhoA/B was only detected in C3-treated CHO cells after 10 min (Figure 7c). The transfected His-tagged RhoA N41I (30 kDa, Figure 7d), which was not ADP-ribosylatable, was only detected by the RhoA-antibody, but not by ADP-ribosylated RhoA/B recognizing antibody. This finding indicates that the new antibody exclusively recognized ADP-ribosylated RhoA/B but not other posttranslational modifications.

**Figure 8:**
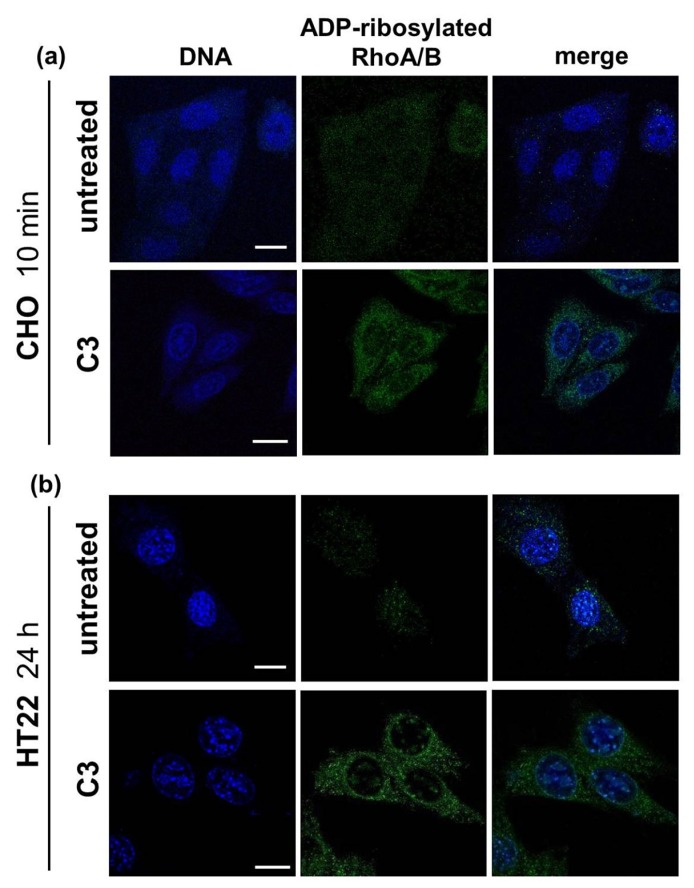
Immunocytochemically analyses of ADP-ribosylated RhoA/B. For imaging ADP-ribosylated RhoA/B, (**a**) CHO cells and (**b**) HT22 cells were incubated with C3 for indicated time points at 37 °C. After washing with phosphate buffered saline (PBS) cells were fixed, permeabilized and incubated with ViF140_A1-hFc antibody following by an Alexa-488 conjugated secondary antibody (green). The nucleus is stained with DAPI (4,6-diamidino-2-phenylindole; blue). Untreated cells were used as control. Cells were imaged by confocal microscopy. Scale bar = 17µm.

To investigate the subcellular localization of ADP-ribosylated RhoA/B, the antibody against ADP-ribosylated RhoA/B (ViF140_A1-hFc) was used in immunocytochemistry. Fluorescence microscopy showed in both cell lines ADP-ribosylated RhoA/B after C3 treatment, whereas untreated CHO (a) and HT22 (b) control cells exhibited weak and diffusely distributed signal (Figure 8). The punctate staining (ADP-ribosylated RhoA/B) was distributed throughout the cytosol. Furthermore, some bright punctuate spots were also found inside the nucleus supporting nuclear localization of ADP-ribosylated RhoA/B.

This finding is consistent with the described cellular distribution of RhoA, which is primarily cytosolic [15,16] but has the capacity to associate with the plasma membrane via a *C*-terminal prenyl group [17,18,19] and a fraction of the total RhoA pool localizes to the nucleus in a GTP-bound active form at steady state [20,21,22]. RhoA colocalized to NF-κB P50 in both cytoplasm and nucleus in human gastric cancer cells [23]. Moreover, LPS appeared to stimulate the nuclear translocation of RhoA protein [24,25]. Thus, ADP-ribosylated Rho was detected in the cytosol and in the nucleus. Membrane-bound ADP-ribosylated Rho was not analyzed.

However, these results confirm the rapid uptake of C3 and the rapid ADP-ribosylation of RhoA/B in CHO cells. In addition, a novel technique to identify and quantify ADP-ribosylated RhoA/B was established and validated against sequential [^32^P]-ADP-ribosylation and gel-shift assay.

## 3. Discussion

As C3 is an established tool in cell biology to study Rho-dependent signaling, the estimation of the cellular amount of Rho inactivated by ADP-ribosylation is essential for conclusion. So far, the amount of intracellular ADP-ribosylated RhoA was determined by different approaches: I) Cell rounding reflects inactivation of cellular RhoA but is improper to quantitate ADP-ribosylated Rho. II) ADP-ribosylation causes an altered migration behavior of RhoA in the SDS-PAGE/Western blot and the shift of modified RhoA to higher apparent molecular weight indicating ADP-ribosylation. This approach is not applicable to all cell lines and is, furthermore, unfortunately characterized by variability and low sensitivity. III) Sequential [^32^P]-ADP-ribosylation of the lysates from C3-treated cells allows only an estimation of Rho ADP-ribosylation as quantification is also variable. Thus, identification of ADP-ribosylated Rho in cell lysates is easy and reliable but exact quantification of ADP-ribosylated Rho is challenging. We generated the monoclonal antibody ViF140_A1-hFc to selectively recognize ADP-ribosylated RhoA/B. ViF140_A1-hFc was characterized to specifically detect ADP-ribosylated RhoA/B but not glucosylated, mutated, or non-modified RhoA. Furthermore, ViF140_A1-hFc recognizes concentration dependently ADP-ribosylated RhoA/B.

This characterized antibody ViF140_A1-hFc was applied to address a surprising observation. C3 seems to be able to ADP-ribosylate RhoA/B in CHO cells within 10 min. This observation was in fact surprising as I. almost complete Rho ADP-ribosylation was not associated with cell rounding and II. all known reports so far referred on at least several hours up to one day delay in the detection of ADP-ribosylated Rho whereby ADP-ribosylation was detected by cell rounding (indirect approach), gel-shift assay or sequential [^32^P]-ADP-ribosylation [7,26]. In CHO cells the SDS-PAGE/Western blot-dependent gel-shift assay did not work but sequential [^32^P]-ADP-ribosylation clearly resulted in decreased labelling reflecting intracellular ADP-ribosylation. Since ADP-ribosylation was not associated with cell rounding a false positive identification of ADP-ribosylated RhoA must be excluded. Therefore, a completely different approach was applied, namely LC-MS analysis using orbitrap MS. As the RhoA peptide harboring the ADP-ribose moiety at Asn-41 is clearly detectable, cell lysates from non-treated and C3-treated cells as well as from cells treated with the enzyme deficient C3 were analyzed by LC-MS. In fact, ADP-ribosylated Rho was only identified in cells treated with C3. In addition, the ADP-ribosylated peptide was also detected by LC-MS after the short treatment period of 10 min. Based on these data we applied the novel antibody ViF140_A1-hFc to determine the uptake of C3 into CHO cells. ViF140_A1-hFc specifically recognized ADP-ribosylated RhoA/B in CHO cells within 10 min of C3 treatment confirming fast cell entry and Rho ADP-ribosylation by C3.

The uptake of C3 into cells seems to depend on the cell type. Presumably, the protein and lipid composition may be responsible for binding and uptake of C3 whereupon the uptake process should be the velocity determining step. Previous studies demonstrated that C3 binds to vimentin that is also involved in the uptake of C3 [8]. CHO cells express vimentin at the cell surface [27,28,29]. However, correlation between cell surface concentration of vimentin and C3-catalyzed ADP-ribosylation has not been studied so far. However, receptor-mediated endocytosis is a mechanism which proceeds in few minutes, e.g., in liver endothelial cells collagen was internalized after 10 s and found in small intracellular structures. During the first 20 min, collagen was detected in early endosomes [30]. Additionally, the mannose receptor is internalized with a half-life of 10 s in rat endothelial cells [31]. In MDCK cells dynamin-dependent endocytosis of occludin was observed at the wound edge within 5 min [32]. Thus, it is conceivable that vimentin-mediated uptake of C3 can take place in this short time period.

In summary, ViF140_A1-hFc antibody specifically recognized ADP-ribosylated RhoA/B and is thus a valuable tool to detect and quantitate ADP-ribosylation of RhoA/B in intact cells. Due to its easy handling it will very probably replace the SDS-PAGE/Western blot-dependent gel-shift and sequential [^32^P]-ADP-ribosylation approaches.

## 4. Materials and Methods

### 4.1. Cell Culture

Wild-type Chinese Hamster Ovary cells (CHO-K1, ATCC: CCL-61) were cultivated in Dulbecco′s modified essential medium/Ham′s F-12 medium (Biochrom GmbH, Berlin, Germany); with 10% fetal bovine serum (Thermo Fisher Scientific, Berlin, Germany), 1% penicillin (Sigma-Aldrich Chemie GmbH, Munich, Germany), 100 units/mL streptomycin (Sigma-Aldrich Chemie GmbH, Munich, Germany), and 1 mM sodium pyruvate (Thermo Fisher Scientific, Berlin, Germany). Cells were maintained at 37 °C and 5% CO_2_. Upon subconfluence, cells were passaged.

Murine hippocampal HT22 cells were cultivated in Dulbecco′s modified essential medium (Biochrom, +10% fetal bovine serum, 1% penicillin, 100 units/mL streptomycin, and 1 mM sodium pyruvate).

### 4.2. Expression and Purification of Recombinant C3 Proteins

C3 wild-type and *Clostridium botulinum*-derived mutant C3-E174Q (carrying a point mutation from glutamate to glutamine at amino acid 174) were expressed as recombinant GST-fusion proteins in *Escherichia coli* TG1 harboring the respective DNA fragment (gene of *Clostridium botulinum* C3, accession No. X59039) in the plasmid pGEX-2T (GE Healthcare Europe GmbH, Freiburg, Germany) and purified by affinity chromatography using glutathione-sepharose. The fusion protein was eluted from the beads using glutathione as described previously [33]. ADP-ribosyltransferase activity was measured by an *in vitro* ADP-ribosylation assay.

### 4.3. Expression and Purification of Recombinant Rho protein

*E. coli* harboring plasmid pGEX2T-RhoA-WT (1-181), pGEX2T-RhoB, pGEX2T-RhoC, pGEX2T-RhoA-N41A and pGEX2T-RhoA-N41I were grown at 37 °C in Luria-Bertani (LB, Becton, Dickinson and Company, New Jersey, NJ, USA) medium containing ampicillin (100 μg/mL; Carl Roth GmbH and Co. KG, Karlsruhe, Germany) to an optical density at 600 nm of 0.8, and isopropyl-β-D-thiogalactopyranoside (IPTG, peqlab Biotechnologie GmbH, Erlangen, Germany) was added to a final concentration of 0.2 mM. The cultures were incubated at 37 °C for an additional 3 h, and the cells were sedimented for 10 min at 4 °C at 7700× *g*, re-suspended in phosphate-buffered saline (PBS) containing 0.1% Triton X-100 (Sigma-Aldrich Chemie GmbH, Munich, Germany), and disrupted by sonication. Cellular debris was sedimented for 10 min at 4 °C at 12,000× *g*, and the resulting supernatant was incubated for 30 min at room temperature with glutathione-sepharose 4B beads (Sigma-Aldrich Chemie GmbH, Munich, Germany) in PBS. The suspension was centrifuged at 500× *g* for 5 min; the pellet was washed five times with PBS. RhoA was released from the parent GST-fusion proteins by incubation with thrombin (GE Healthcare Europe GmbH, Freiburg, Germany, 10 NIH units/mL of bead suspension) at 4 °C overnight in buffer (150 mM NaCl, 5 mM MgCl_2_, 2.5 mM CaCl_2_ , 1 mM dithiothreitol, and 50 mM Tris-HCl, pH 8.0). The beads were removed by centrifugation for 10 min at 500× *g*, and thrombin was precipitated using p-aminobenzamidine beads (Sigma-Aldrich Chemie GmbH, Munich, Germany). The homogeneity of the recombinant RhoA was proven by SDS-PAGE.

### 4.4. Western Blot Analysis

Western blot analyses were performed as described previously [7]. Briefly, complete lysate proteins were separated using 15% SDS-PAGE and subsequently transferred onto nitrocellulose membranes (GE Healthcare UK Limited, Buckinghamshire, UK) and blocked with 5% (*w*/*v*) non-fat dried milk (Sucofin TSI GmbH, Zeven, Germany). For Western blot analysis the following primary antibodies were used: RhoA was identified using a mouse monoclonal IgG from Santa Cruz Biotechnologies sc-418 (Santa Cruz, CA, USA) and Actin (Sigma-Aldrich Chemie GmbH, Munich, Germany) was used as loading control. For the chemiluminescence reaction, ECL Femto (Pierce, Thermo Fisher Scientific Inc., Rockford, IL, USA) was used. All signals were analyzed densitometrically using the KODAK 1D software (Version 3.5, KODAK GmbH, Stuttgart, Germany, 2001) and normalized to β-actin signals.

### 4.5. ADP-Ribosylation of Rho in Murine Cells

To verify the effectiveness of ADP-ribosylation of Rho by *C. botulinum* exoenzyme C3, the cultivated cells were either left untreated or were incubated with 500 nM C3 for indicated time points. The cells were then washed with PBS and scraped into 100 µL of lysis buffer (20 mM Tris⁄HCl (pH 7.4), 1% Triton X-100, 10 mM NaCl (J.T.Baker/Avantor Performance Materials, Center Valley, PA, USA), 5 mM MgCl_2_ (Merck KGaA, Darmstadt, Germany), 1 mM phenylmethanesulfonyl fluoride (Serva Electrophoresis GmbH, Heidelberg, Germany), 5 mM dithiothreitol (Serva Electrophoresis GmbH, Heidelberg, Germany)). The obtained suspension was shaken at 37 °C for 10 min. Ultrasonic disruption was performed using a cycle of 10 × 5 s, 5 × 10% sonic energy with a sonotrode (Bandelin Electronic, Berlin, Germany). Protein concentrations were measured by the Bradford method. Cell lysates containing equal amounts of protein were incubated with 1000 nM recombinant *C. botulinum* exoenzyme C3 and 1 µCi [^32^P]-NAD (Amersham Life Sciences, Arlington Heights, IL, USA) in 20 µL of buffer containing 50 mM HEPES (Serva Electrophoresis GmbH, Heidelberg, Germany) (pH 7.3), 10 mM MgCl_2_, 10 mM dithiothreitol, 10 mM thymidine (Sigma-Aldrich Chemie GmbH, Munich, Germany) and 10 µM NAD (Serva Electrophoresis GmbH, Heidelberg, Germany) at 37 °C for 20 min. The reaction was terminated by addition of Laemmli sample buffer, and then incubated at 95 °C for 10 min. Samples were resolved by SDS-PAGE on 15% gels, and the ADP-ribosylated Rho was analyzed by phosphorimaging (Cyclone, Packard American Instrument, Haverhill, MA, USA). The SDS-PAGE gel was stained with Coomassie brilliant blue 250 (Sigma-Aldrich Chemie GmbH, Munich, Germany).

### 4.6. ADP-Ribosylation of Recombinant Rho Proteins

250 ng of recombinant Rho proteins were incubated with 500 nM C3 in 20 µL of buffer containing 50 mM HEPES (pH 7.3), 10 mM MgCl_2_, 10 mM dithiothreitol, 10 mM thymidine at 37 °C for 30 min. The reaction was terminated by addition of Laemmli sample buffer, and then incubated at 95 °C for 10 min. Samples were separated by SDS-PAGE on 15% gels, and the ADP-ribosylated Rho was analyzed by Western blot with RhoA and ADP-ribosylated RhoA specific antibody.

For ADP-ribosylation of RhoB and RhoC, 500 ng of recombinant Rho proteins were incubated with 500 nM C3 and proceeded as described above. A second SDS-PAGE gel was run in parallel and stained with Coomassie brilliant blue 250.

### 4.7. Glucosylation of Recombinant Rho Proteins

250 ng of recombinant Rho proteins were incubated with 150 ng/µL TcdB in 20 µL of buffer containing 50 mM HEPES (pH 7.3), 10 mM MgCl_2_, 10 mM dithiothreitol, 10 mM thymidine at 37 °C for 30 min. The reaction was terminated by addition of Laemmli sample buffer, and then incubated at 95 °C for 10 min. Samples were separated by SDS-PAGE on 15% gels and subsequently analyzed in Western blot against RhoA and ADP-ribosylated RhoA.

### 4.8. Transfection of CHO Cells

The purified RhoA N41I cDNA was digested with *Nco*1 and *Xho*1 and cloned into pQE-TriSystem plasmid vector (Qiagen, Valencia, CA, USA). Plasmid DNA of RhoA N41I and EGFP were prepared using the plasmid miniprep kit (peqlab Biotechnology GmbH, Erlangen, Germany) according to manufacturers’ instructions. CHO cells were plated at 2.5 × 10^5^ onto 3.5 cm plates and grown for 24 h at 37 °C and 5% CO_2_. The medium was removed and cells were washed with PBS. Cells were transfected with 2 μg plasmid DNA construct of His-tagged RhoA N41I or transfected with pQE-TriSystem-EGFP by use of jetPrime Polyplus transfection system (Polyplus transfection S.A., Illkirch, France) according to manufacturers’ instructions. After 48 h the cells were treated with 500 nM C3 for 10 min. After this incubation, cells were washed with PBS and scraped into Laemmli sample buffer.

### 4.9. LC-MS Analysis

CHO cells were treated with 500 nM C3 or 500 nM C3-E174Q for 10 min. Untreated cells served as control. Cells were washed and scraped into Laemmli sample buffer and lysed. The lysate was then incubated at 95 °C for 10 min followed by cysteine modification with acrylamide (Serva Electrophoresis GmbH, Heidelberg, Germany) for 20 min at room temperature. Samples were submitted to SDS-PAGE and stained with PageBlue Protein staining solution (Thermo Fisher Scientific, Berlin, Germany). Protein bands between 20 and 30 kDa were manually excised and cut into 1 mm^3^ cubes. The destaining, dehydration, digestion, and peptide extraction were performed as described before [12].

Peptides were dissolved in 30 µL 2% (*v*/*v*) ACN (Carl Roth GmbH and Co. KG, Karlsruhe, Germany), 0.1% (*v*/*v*) TFA (Merck KGaA, Darmstadt, Germany). After centrifugation for 2 min at 13,500 *g*, the supernatant was transferred to an LC sample vial and an appropriate amount of each sample was injected into the LC system (Ultimate 3000 RSLC system, Dionex, Sunnyvale, CA, USA). Peptide separation was performed as described previously [12]. The LC system was online connected to the nanoESI source of an LTQ Orbitrap velos (Thermo Fisher Scientific, Dreieich, Germany). MS precursor scans were acquired from 300–1700 *m*/*z* in profile mode with a resolution of 60,000 at 400 *m*/*z* in the orbitrap mass analyzer. The top 10 most intense ions of charge state +2 and +3 and a minimum signal threshold of 1800 counts were selected for CID fragmentation with normalized collision energy of 38%, an activation time of 10 ms and activation Q of 0.250. The masses of the tryptic ADP-ribosylated and unmodified peptide of RhoA was set on an inclusion list for preferred fragmentation. Fragments were scanned out in the ion trap with normal scan rate. Data were analyzed with Proteome Discoverer 1.4 (Thermo Fisher Scientific, Dreieich, Germany). Spectra were searched with Mascot search algorithm and the uniprot hamster database with maximum of two missed cleavage sites and, propionamidation of cysteine, oxidation of methionine, acetylation of lysine and ADP-ribosylation of asparagine as dynamic modifications. Peptide mass tolerance was set to ±4 ppm and fragment mass tolerance to 0.8 Da.

### 4.10. Generation of A Specific Antibody Recognizing ADP-Ribosylated RhoA

Recombinant RhoA (50 µg/mL) was subjected to ADP-ribosylation by glutathione-sepharose 4B beads conjugated GST-C3 exoenzyme (75 µg/mL) in 400 µL of buffer containing 50 mM HEPES (pH 7.3), 50 mM MgCl_2_, 25 mM dithiothreitol, 100 mM thymidine, 25 µM NAD, and 100 mM GDP (Sigma-Aldrich Chemie GmbH, Munich, Germany) for 60 min at 37 °C and 400 rpm. Beads conjugated GST-C3 was removed by centrifugation at 15,600× *g* for 5 min. The resulting supernatant with the ADP-ribosylated RhoA was analyzed by SDS-PAGE.

Antibodies against ribosylated RhoA were selected from the human naive antibody gene libraries HAL9/10 [14]. The selection and screening was performed as described before [13]. Before antibody selection the library was pre-incubated on non-ADP-ribosylated RhoA immobilized on Costar High Binding microtiter plates (Sigma-Aldrich Chemie GmbH, Munich, Germany). After pre-incubation the scFv phage supernatant were transferred on ADP-ribosylated RhoA immobilized on Costar High Binding microtiter plates for antibody selection. After three rounds of Panning 94 clones were screened for production of RhoA binding scFvs by antigen-ELISA. After identification of scFv producing clones plasmid DNA was isolated and the antibody DNA was sequenced. Identified antibody-producing cells were stored as a glycerol stock in a −80 °C freezer.

### 4.11. Production of scFv-Fc (Yumabs)

The scFv was recloned into pCSE2.6-hIgG1-Fc-XP using *NcoI*/*Not*I for mammalian production as scFv-Fc (Yumab), an IgG-like antibody format. The production and purification was performed as described before [34].

### 4.12. Immunocytochemistry

HT22 and CHO cells seeded on cover slips were washed with PBS and subsequently fixed in 4% paraformaldehyde in phosphate buffered saline (PBS) (pH 7.4) at room temperature for 20 min. Cells were then washed and permeabilized with 0.3% (*w*/*v*) Triton X-100 (Sigma-Aldrich Chemie GmbH, Munich, Germany) in PBS supplemented with 5% BSA (Sigma-Aldrich Chemie GmbH, Munich, Germany). ADP-ribosylated Rho was stained by ViF140_A1-hFc antibody and Alexa-488 conjugated secondary antibody for 1 h at room temperature either. Then, a 0.1 µg/mL solution of DAPI (Sigma-Aldrich Chemie GmbH, Munich, Germany) in phosphate-buffered saline supplemented with 0.1% (*w*/*v*) Tween-20 (Serva Electrophoresis GmbH, Heidelberg, Germany) was used for nuclei staining for 30 min at 37 °C. Cells were analyzed using a Leica TCS 2 inverted confocal microscope (Leica Mikrosysteme Vertrieb GmbH, Wetzlar, Germany).

### 4.13. Confocal Laser Scanning Microscopy

For image acquisition a Leica TCS 2 SL confocal laser scanning microscope (Leica Mikrosysteme Vertrieb GmbH, Wetzlar, Germany) using a 63× oil immersion objective was used. Fluorescent dyes were excited at a wavelength of 488 nm (green fluorescence) and 405 nm (blue fluorescence), respectively. Images were captured at a resolution of 1024 × 1024 pixels (Leica Confocal Software, Lite Version 2.5, Leica Mikrosysteme Vertrieb GmbH, Wetzlar, Germany, 2004).

### 4.14. Reproducibility of the Experiments and Statistics

All experiments were performed independently at least three times. Results from representative experiments are shown in the figures. Statistical significance was calculated with GraphPad Prism software (Version 6, GraphPad Software, Inc., San Diego, CA, USA, 2013) utilizing a Student’s *t*-test. Values (*n* ≥ 3) are means ± SEM. Differences were considered to be statistically significant at *p* ≤ 0.05 (* = *p* ≤ 0.05, ** = *p* ≤ 0.01 and *** = *p* ≤ 0.001).

## Figures and Tables

**Table 1:** Detection of ADP-ribosylated RhoA by mass spectrometry (MS). Identification of ADP-ribosylated RhoA in C3-treated Chinese hamster ovary (CHO) cells and unmodified RhoA are depicted.

Identified Peptide	Identified Modification	Calculated *m*/*z* of the Peptide	C3			C3-E174Q			Control		
Peptide Sequence	Modification	MH+ (Da)	IonScore	*m*/*z* (Da)	RT (min)	IonScore	*m*/*z* (Da)	RT (min)	IonScore	*m*/*z* (Da)	RT (min)
DQFPEVYVPTVFENYVADIEVDGK	-	2773	75	1387	157	78	1387	157	80	925	157
DQFPEVYVPTVFEnYVADIEVDGK	N14(ADP-Ribosyl)	3314	31	1105	169	-	-	-	-	-	-

## Abbreviations

The following abbreviations are used in this manuscript:

C3*bot*	C3 exoenzyme derived from *Clostridium botulinum*
SDS	sodium dodecyl sulfate
PAGE	polyacrylamide gel electrophoresis
LC-MS/MS	Liquid chromatography–tandem mass spectrometry
